# A Sporadic Case of *COL1A1* Osteogenesis Imperfecta: From Prenatal Diagnosis to Outcomes in Infancy—Case Report and Literature Review

**DOI:** 10.3390/genes14112062

**Published:** 2023-11-10

**Authors:** Karolina Vankevičienė, Aušra Matulevičienė, Eglė Mazgelytė, Virginija Paliulytė, Ramunė Vankevičienė, Diana Ramašauskaitė

**Affiliations:** 1 Faculty of Medicine, Vilnius University, M. K. Čiurlionio Str. 21/27, LT-03101 Vilnius, Lithuania; 2 Department of Human and Medical Genetics, Institute of Biomedical Sciences, Faculty of Medicine, Vilnius University, Santariskiu Str. 2, LT-08661 Vilnius, Lithuania; ausra.matuleviciene@mf.vu.lt; 3 Department of Physiology, Biochemistry, Microbiology and Laboratory Medicine, Institute of Biomedical Sciences, Faculty of Medicine, Vilnius University, M. K. Čiurlionio Str. 21/27, LT-03101 Vilnius, Lithuania; egle.mazgelyte@mf.vu.lt; 4 Clinic of Obstetrics and Gynaecology, Institute of Clinical Medicine, Faculty of Medicine, Vilnius University, M. K. Čiurlionio Str. 21/27, LT-03101 Vilnius, Lithuania; virginija.paliulyte@mf.vu.lt; 5 Clinic of Children’s Diseases, Institute of Clinical Medicine, Faculty of Medicine, Vilnius University, M. K. Čiurlionio Str. 21/27, LT-03101 Vilnius, Lithuania; ramune.vankeviciene@mf.vu.lt

**Keywords:** bone fractures, brittle bone disease, bisphosphonate, *COL1A1* variant, fetal fractures, osteogenesis imperfecta, prenatal ultrasound screening, skeletal dysplasia

## Abstract

Osteogenesis imperfecta (OI), also known as brittle bone disease, belongs to a rare heterogeneous group of inherited connective tissue disorders. In experienced prenatal centers, severe cases of OI can be suspected before birth from the first trimester prenatal ultrasound screening. In this article, we describe a case report of OI suspected at the 26th week of gestation and the patient’s outcomes in infancy one year after birth, as well as compare our case to other prenatally or soon-after-birth suspected and/or diagnosed OI clinical case reports in the literature. This case was managed by a multidisciplinary team. In this clinical case, OI was first suspected when prenatal ultrasound revealed asymmetric intrauterine growth restriction and skeletal dysplasia features. The diagnosis was confirmed after birth using *COL1A1* gene variant detection via exome sequencing; the *COL1A1* gene variant causes OI types I–IV. The familial history was negative for both pregnancy-related risk factors and genetic diseases. At one year old, the patient’s condition remains severe with bisphosphonate therapy.

## 1. Introduction

Osteogenesis imperfecta (OI), also known as brittle bone disease, is a rare heterogeneous group of inherited connective tissue disorders [1]. The group of disorders is subdivided into collagen and non-collagen OI types and further classified into types from I to XXI or unclassified. Types I to IV belong to collagen OI, whereas types V to XXI belong to the non-collagen OI type. Types I to IV account for 85–90% of cases and occur due to variants in the *COL1A1* or *COL1A2* genes encoding type I collagen, which is responsible for the bone, skin, and tendon extracellular matrices. The non-collagen and unclassified types account for the remaining 10–15% of cases and are caused by gene variants encoding the proteins that interact with collagen [2,3,4,5]. OI is a rare disorder and its various types are diagnosed in 1 in 15,000–20,000 pregnancies [1]. OI can be inherited in autosomal dominant, autosomal recessive, or X-linked recessive manners [6]. However, 60% of single occurrences in family cases are caused by a de novo variant or an inherited pathogenic variant from a parent with somatic and/or germline mosaicism. Mosaicism can be present in up to 16% of families [7]. As the primary defect affects type I collagen, this generalized connective tissue disorder has a major clinical impact on the skeletal system. However, the clinical features and disease severity depend on the disease type. High bone fragility, easy fractures following minimal or no trauma, bone deformities, and motor, development, and growth delay are usually observed as the primary clinical features. Other features depend on the OI type and can include blue sclera, dentinogenesis imperfecta, joint laxity, low muscle tone, hearing impairment including hearing loss due to abnormalities affecting middle and/or inner ears, life-threatening respiratory insufficiency due to underdeveloped lungs and thorax deformations, and congestive heart failure [7,8]. In experienced centers, the lethal and most severe OI forms can be suspected during prenatal ultrasound examination from the first trimester of pregnancy, whereas milder forms can be suspected later in pregnancy if any skeletal dysplasia features occur [9]. In this article, we present a case report of *COL1A1* gene c.3150_3158dup p.(Ala1053_Gly1055dup) (rs74315111) variant OI that leads to type I–IV OI suspected in the 26th week of pregnancy and the patient’s outcomes one year after birth; we also present a review of other case reports of OI suspected during the prenatal period or soon after birth. From the suspicion of the diagnosis, this clinical case was further managed with a multidisciplinary team.

## 2. Case Report

### 2.1. Prenatal Findings and Further Pregnancy Care

A 26-year-old primigravida woman was referred to the tertiary-level hospital for a geneticist’s consultation in the Prenatal Diagnostic Unit of the Center for Medical Genetics due to suspected fetal intrauterine growth restriction at 26 weeks of gestation, detected during a routine antenatal care visit.

**Figure 1:**
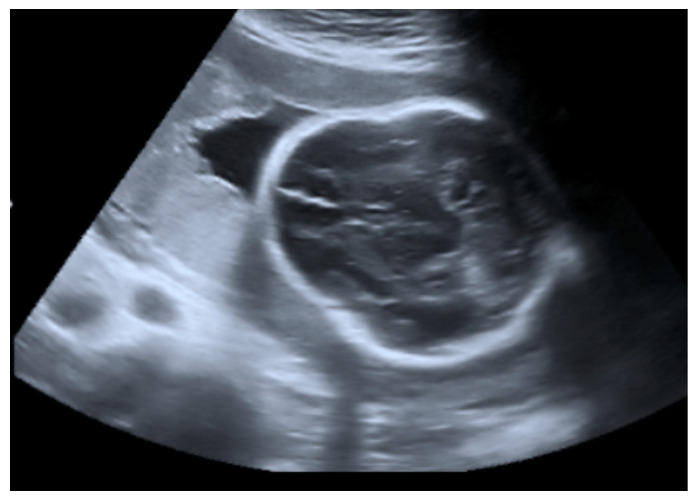
Transabdominal fetal ultrasound scan image including an axial view of the fetal head in the transthalamic plane. A deformed skull contour was observed.

**Figure 2:**
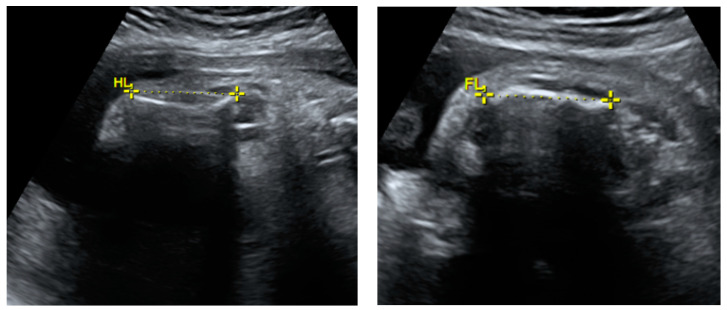
Transabdominal fetal ultrasound scan images including fetal long bones. Deformed and shortened long bones (humerus and femur) were observed. HL—humerus length; FL—femur length.

**Figure 3:**
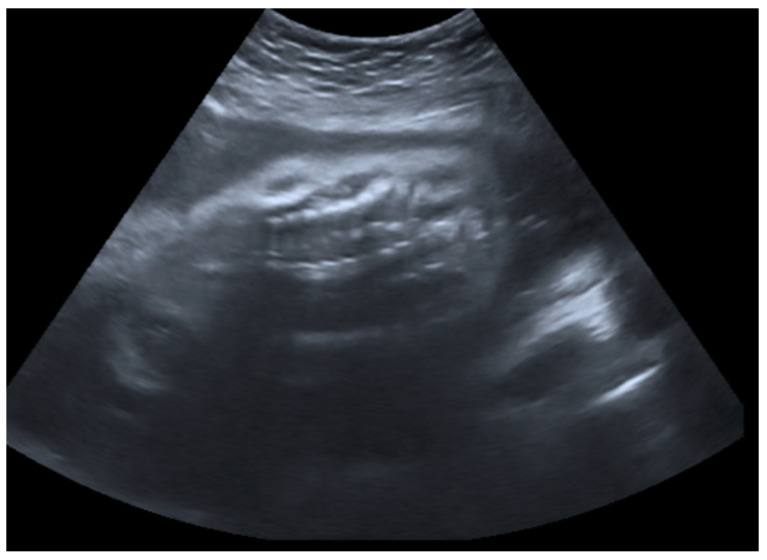
Transabdominal fetal ultrasound scan image including a coronal view of the fetal spine and chest. A deformed and compressed chest was observed.

The fetal ultrasound in the experienced prenatal care center at 26 weeks of gestation revealed a normal amount of amniotic fluid, intrauterine growth restriction, and fetal skeletal dysplasia features including a deformed skull (Figure 1), deformed and shortened long bones (Figure 2), deformed and compressed chest cavity (Figure 3), and broken ribs and humerus. At 26 + 4 weeks of gestation, the growth of the fetus was equivalent to 22 + 6 weeks of gestation and asymmetric-type intrauterine growth restriction (<3rd percentile) was diagnosed. Based on the ultrasound examination findings, skeletal dysplasia (OI or thanatophoric dysplasia) was suspected.

Genetic counseling of the parents revealed that the mother’s height was 165 cm and weight was 66 kg and the father’s height was 178 cm and weight was 75 kg. The father was 27 years old. During consultation, both parents’ phenotypes were described as normal. There were no risk factors for pregnancy. The parents denied both genetic diseases in family and familial histories for skeletal disorders. The woman underwent amniocentesis at 26 weeks of gestation. Cytogenetic analysis revealed a fetus karyotype of 46,XY. The results of an SNP comparative genomic hybridization assay were normal. To rule out suspected thanatophoric dysplasia, fibroblast growth factor receptor 3 (*FGFR3*) gene sequencing from the gDNA extracted from amniocytes was performed. The results showed no clinically significant variants in the *FGFR3 gene*.

For the rest of the pregnancy, the condition and well-being of the mother and the fetus were monitored during antenatal visits to the perinatology center. Due to the unfavorable prognosis, multiple fetal anomalies, skeletal dysplasia, breech position of the fetus, and a family request, the multidisciplinary team recommended giving birth by a planned Cesarean section at 39 weeks of gestation. After the delivery, immediate genetic consultation and examination of the newborn was recommended. 

### 2.2. Birth and Early Treatment

As planned, a Cesarean section was performed at 39 weeks of gestation. A male newborn was born, weighing 2490 g (<3rd percentile) and at 44 cm height. The Apgar scores were 6 and 8 after 1 and 5 min, respectively. The pH value of the umbilical artery was 7.43. Due to severe respiratory failure, the initial steps of newborn resuscitation were performed and mechanical lung ventilation using a mask was started; however, this was not effective. Therefore, the newborn was intubated and mechanical lung ventilation was started through the intubation tube. For further investigations and treatment, the newborn was transported to the Neonatal Intensive Care Unit (NICU). The treatment plan included mechanical lung ventilation, antimicrobial therapy, infusion therapy, parenteral nutrition, and adequate analgesia. 

**Figure 4:**
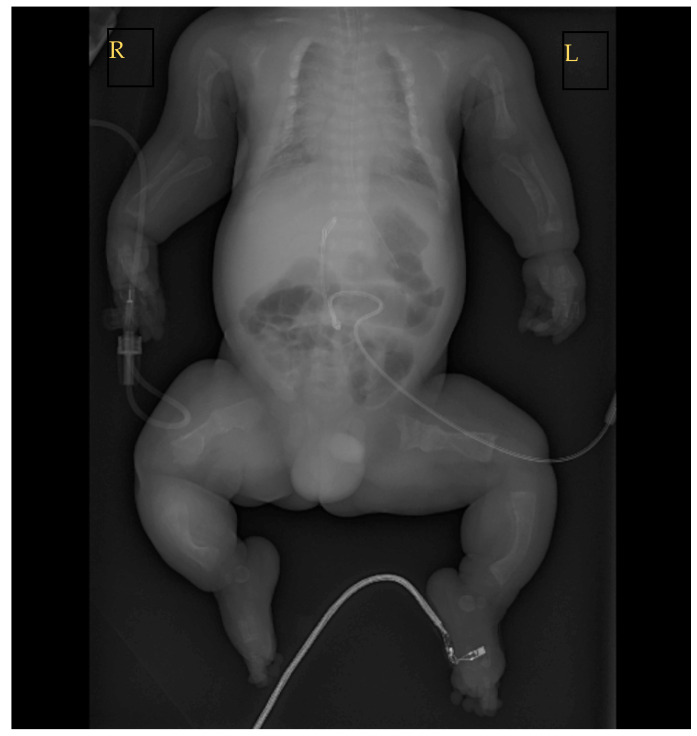
Chest, abdomen, and extremities X-ray of the lying newborn. Conclusions of the X-ray included: signs of skeletal dysplasia—multiple bone deformities with possible previous bone fractures in utero; fracture of the right ulna in the middle third; and a deformed and narrow chest cavity with incompletely expanded lungs. R—right side, L—left side.

The patient underwent head, chest, abdomen, and extremities X-rays. Multiple bone deformities and bone fractures present before birth were confirmed radiologically (Figure 4). Due to the fracture in the middle of the right ulna, it was decided to place a cast.

**Figure 5:**
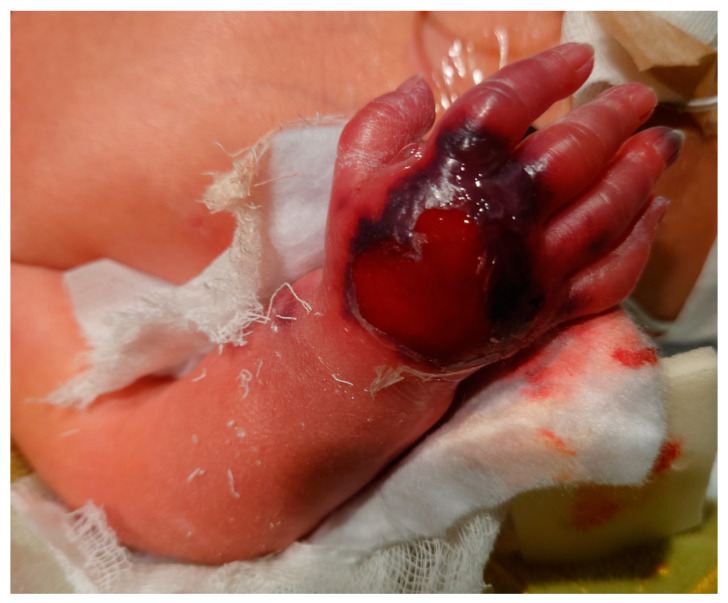
Right hand epidermal necrosis observed after cast removal.

The next day, impaired microcirculation of the right hand was noticed. The cast was removed. Signs of epidermal necrosis were observed (Figure 5). It was decided to remove the cast and start treating the wound conservatively.

### 2.3. Further Patient Investigations, Management, and Treatment after Birth

Three days after birth, the patient was transferred to the tertiary-level Children’s hospital. The patient’s care and treatment were continued using a multidisciplinary team approach. The team included neonatologists, geneticists, orthopedists, general surgeons, occupational therapists, ophthalmologists, cardiologists, pediatricians, and rare diseases, palliative care, and pain specialists. The care after birth was focused on optimizing the patient’s respiratory status, minimizing discomfort and pain during routine care and procedures, and reducing the risk of new fractures. 

The patient’s hospitalization lasted 77 days from birth. During this period, the patient was hospitalized in the NICU two times—first after birth and then one time when the patient’s condition worsened in the neonatal care unit. For one month he was hospitalized in the neonatal care unit; for the remaining period, after using all the possible active treatment opportunities and due to the remaining severe general condition and respiratory distress symptoms’ requiring the need of high-flow oxygen therapy via nasal cannulas, he was treated in the Children’s Pain and Palliative Care Clinic unit.

During hospitalization, the patient’s treatment focused on respiratory insufficiency treatment with mechanical lung ventilation through an intubation tube; after the extubation treatment was continued via high-flow nasal cannula oxygen therapy. Signs of respiratory distress such as tachypnea and involvement of the intercostals were constantly observed. Antibacterial treatment, beta-blockers, diuretics, infusion therapy, adequate analgesic treatment, and vitamin D supplements were applied. Superficial skin lesions, including epidermal necrosis of the right hand, were treated conservatively with daily wound dressings and have healed completely. Feeding included mother’s milk, adapted milk mixture, and parenteral feeding when necessary. Prevention of hemorrhagic newborn disease was achieved using vitamin K and vaccinations according vaccination schedule were administered. The multidisciplinary team decided to start treating the patient with bisphosphonates. A three-day intravenous treatment course of Pamidronate was prescribed every two months up to 2 years of age. 

After 77 days of hospitalization, the patient was discharged home with high-flow nasal cannula oxygen therapy and prescribed medical treatment including regular bisphosphonate treatment courses in the hospital with outpatient family doctor’s supervision and follow up at The Center for Coordination of Rare Diseases.

From the discharge home, the patient was hospitalized and treated in the Children’s hospital two more times due to aggravated chronic respiratory insufficiency. His worsened conditions requiring hospitalizations were due to pneumonia and due to COVID-19 disease.

### 2.4. Follow-Up and Outcome One Year after Birth

At 1 year old, the child’s condition is stable with bisphosphonate therapy but remains severe. The treatment with bisphosphonates is well tolerated. No new fractures have been diagnosed since the beginning of the treatment.

The prescribed medical treatment, outpatient and palliative care, nursing services, and physiotherapy is provided and continued constantly. Oxygen therapy via high-flow nasal cannulas is continued. Due to insufficient nutrition and swallowing problems, the child is additionally fed through a nasogastric tube. 

At the age of 1, growth, development, and motor delay are observed. Both height and weight are below the third percentile. Currently, the child is communicative and reacts to the sounds of toys and people around him. The child’s limbs are deformed; his muscles are hypotonic and hypotrophy is observed. His movements are limited. He is able to sit with support and keeps his head up briefly when lying in a prone position. However, assistance and help are needed in all active actions.

### 2.5. Genetic Counseling

After birth, the patient was immediately examined by a geneticist. During examination, changes in the phenotype were observed (Table 1). 

To make an accurate diagnosis in light of the suspicion of skeletal dysplasias, whole-exome sequencing was performed. Whole-exome sequencing revealed a heterozygous pathogenic variant of the *COL1A1* gene c.3150_3158dup p.(Ala1053_Gly1055dup) (rs74315111). The identified variant results in the insertion of three amino acids (p.Ala1053_Gly1055dup) in the N-terminal part of the triple helix of type I collagen and is predicted to add one canonical Gly-X-Y repeat unit. The variant was not found in the Genome Aggregation Database (gnomAD) and was predicted to be pathogenic using bioinformatics tools. Short in-frame deletions or duplications of three, nine, or 18 nucleotides in the triple-helical region of the *COL1A1* gene cause OI [10]. 

**Figure 6:**
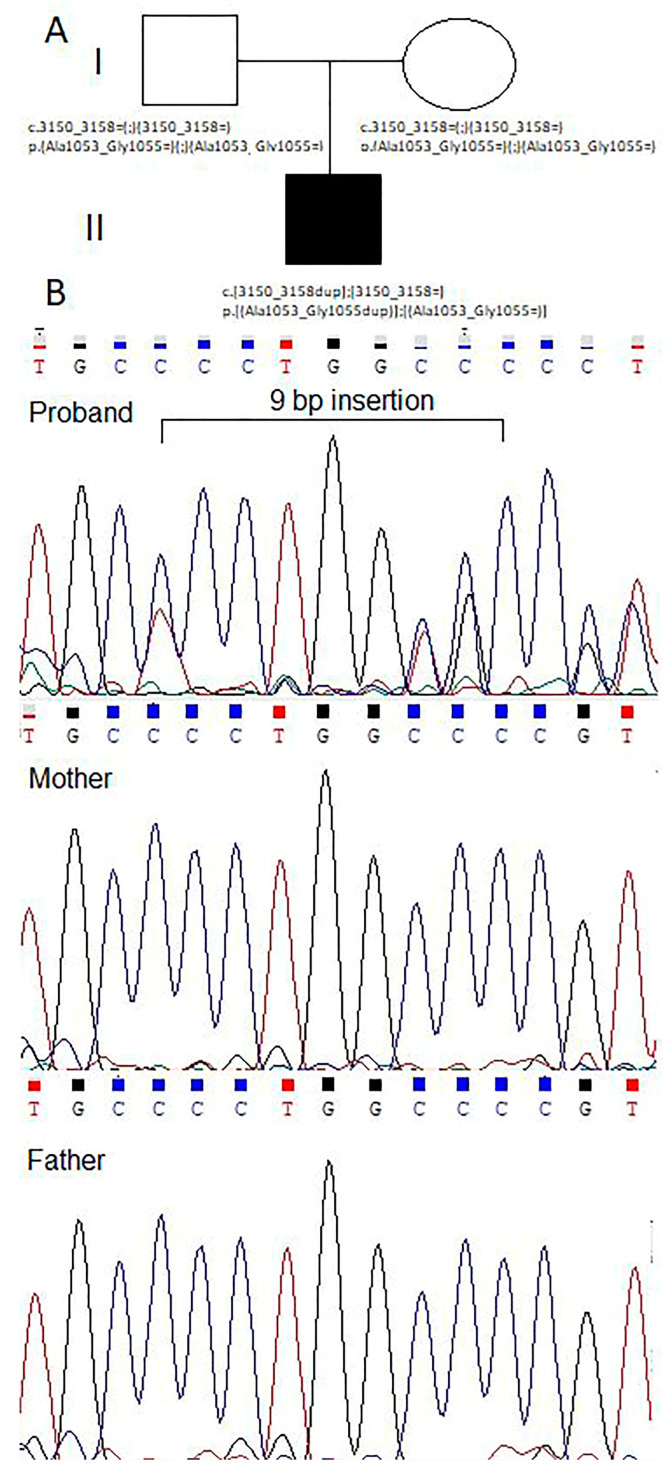
(**A**) The genealogy of the family presenting affected proband and healthy parents. (**B**) Sanger sequencing reads demonstrating the *COL1A1* variant c.3150_3158dup in the proband gDNA sample.

To evaluate the recurrence risk in the family, segregation analysis using the Sanger sequencing method for the aforementioned *COL1A1* variant in the family was performed. The *COL1A1* gene c.3150_3158dup p.(Ala1053_Gly1055dup) (rs74315111) variant was confirmed in the heterozygous state in the gDNA sample of the proband and was not present in either of the parents, suggesting it was de novo (Figure 6). The risk of recurrence of OI in the family is 5–6%, but somatic and/or germline mosaicism cannot be ruled out.

## 3. Discussion and Literature Review

Prenatal ultrasound examination is considered as an effective tool for the suspicion and diagnosis of skeletal dysplasia, including OI. If skeletal dysplasia is severe, it is possible to diagnose it from the first pregnancy trimester. The ultrasound features of a severe skeletal dysplasia in the first trimester can include increased nuchal translucency, a small crown–rump length for gestational age, deformed and shortened limbs, a small chest and ribs, the hypoechoic appearance of the affected bones due to the decreased bone density and mineralization, and fractures sustained in utero [11,12]. During the second trimester ultrasound scan, any fetus showing long bone measurements of less than the fifth percentile should be evaluated in a specialized center with the possibility consultation with a geneticist [13]. Other nonspecific features such as polyhydramnios may be present due to the reduced fetal swallowing caused by skeletal abnormalities involving the chest cavity [14]. However, accurate prenatal diagnosis of skeletal dysplasias remains problematic. The sensitivity of diagnosing a correct skeletal dysplasia during a routine 2D ultrasound reaches only 60% accuracy [15]. Accuracy in predicting the lethality can reach 100%. The signs of lethality during an ultrasound examination include early severe shortening of the long bones (<1st percentile), a diminished femur-length-to-abdominal-circumference ratio, hypoplasia of the thorax, short ribs, marked bone bowing or fractures, caudal regression, and a cloverleaf-shaped skull [9]. It is important to detect skeletal dysplasia and distinguish between the lethal and nonlethal forms in order to offer respectful patient counseling to either terminate or continue with the pregnancy according to the gestational age and pregnancy termination options available in the country. Furthermore, the prenatal detection of skeletal dysplasia allows for the preparation of further patient care prior to the delivery and a treatment plan after birth.

In our case, the prenatal diagnosis of OI was complicated. Intrauterine growth restriction (IUGR) was first suspected at the 26th week of pregnancy during an antenatal care visit. First and second trimester ultrasound protocols were described without pathological findings. This can be the case when skeletal dysplasia is not lethal and changes can be found during later ultrasound examinations. After finding IUGR, the patient was immediately referred for a more detailed ultrasound examination at a tertiary-level perinatology center with the possibility of a geneticist’s counseling After a detailed ultrasound examination, skeletal dysplasia, possibly OI, was suspected. In order to rule out thanatophoric dysplasia, fibroblast growth factor receptor 3 (*FGFR3*) gene sequencing from the gDNA extracted from amniocytes was performed. The results showed no clinically significant variants in the *FGFR3* gene. Whereas most individuals diagnosed with thanatophoric dysplasia die in the perinatal period due to complications, once the pathogenetic variant is identified, patient care tactics after birth can focus on providing comfort care [16]. When thanatophoric dysplasia was ruled out from the differential diagnoses, it was decided to make an accurate diagnosis after birth with whole-exome sequencing. However, the suspicion of skeletal dysplasia, even without a molecular diagnosis (OI was confirmed only after birth), allowed patient care planning prior to the delivery of the newborn.

In order to compare our case with others, a search for clinical cases using an advanced search in PubMed including the keywords “osteogenesis imperfecta”, “brittle bone disease”, “skeletal dysplasia”, “neonate”, “prenatal”, “antenatal”, and “ultrasound” was performed. Case reports published in English within the most recent five-year period with suspected OI before or shortly after birth were included. The main clinical case report information is presented in Table 2.

In the reviewed clinical cases, the earliest suspicion of OI was from the 17th week of gestation. One more case was suspected in the second trimester of pregnancy; the remaining were at the end of the pregnancy or only after birth. This confirms that the diagnosis of skeletal dysplasia remains difficult, especially if the signs visible during an ultrasound are minimal and suggest a non-lethal OI type. The earlier OI is suspected, the worse the prognosis is likely to be. In our clinical case, although the prognosis for the fetus was not favorable, the pregnancy was continued because of the gestational age. From the reviewed clinical cases, when OI was suspected at 17 weeks of gestation, due to the unfavorable prognosis for the fetus, the parents chose pregnancy termination.

When there is a familial history of OI, genetic counseling and diagnosis are offered to the expectant parents. When family history is negative for genetic diseases, skeletal dysplasia including OI is usually suspected when the femur length is decreased in the second trimester of pregnancy [25]. In familial OI cases, genetic testing can confirm the diagnosis. Invasive prenatal testing methods, including chorionic villus sampling (CVS) and amniocentesis, are the most commonly used techniques. Molecular analysis testing using DNA extracted from CVS cells or amniocytes is possible for all types of OI if the variant in the family is known [26]. In this clinical case, as well as in the reviewed clinical cases, there was no history of familial OI. In our clinical case and the cases where OI was suspected before birth, short and bowed long bones were detected during ultrasound examination. In our clinical case, OI before birth was not confirmed. According to the gestational age (26 weeks), amniocentesis was performed, and cytogenetic analysis revealed a fetus karyotype of 46, XY; comparative genomic hybridization was normal. OI was confirmed only after birth when a whole-exome sequencing was performed. Out of the reviewed case reports, OI was confirmed in all but one. Betoko et al. [21] pointed out that expensive genetic tests are still not available in low-income countries, leading to diagnoses that are based only on the clinical findings. 

In our clinical case, the newborn was delivered by a planned Cesarean section (CS). According to research, in cases of OI the birth fracture rates do not differ between vaginal and CS deliveries, and CS is not associated with decreased fracture rate. However, fractures in utero, a maternal history of OI, and breech presentation are strong predictors for choosing CS delivery. Therefore, CS delivery is recommended to be performed only if there are other maternal or fetal indications, not solely OI [27]. In this clinical case, the CS was chosen not only because of OI but also because of poor fetal prognosis, fractures in utero, breech fetal position, and family request. 

For the optimal management of patients diagnosed with OI, a multidisciplinary team involving a wide list of health specialists is necessary. The primary care should focus on maintaining respiratory function, reducing pain, and improving motor function [28]. In our clinical case, the patient was managed by a multidisciplinary team from the suspicion of the OI diagnosis before birth. 

The most common OI-associated morbidity and mortality causes after birth include cardiopulmonary insufficiency and respiratory infections due not only to secondary problems arising from thoracic skeletal dysplasia, including scoliosis and rib cage deformities of different severities, but also to intrapulmonary collagen alterations. Since OI-determined thoracic deformities and fractures result in structural abnormalities of the chest wall and spine, thus limiting the pulmonary function, it is recommended to pay routine attention to the spine, prevent new fractures, and correct bone deformities. Furthermore, it is recommended to monitor pulmonary function even when thoracic deformities are absent because this is likely not the only cause of pulmonary disease in OI patients. Thus, it is recommended to treat difficulties in breathing and respiratory infections immediately when suspected to prevent cardiopulmonary complications [29]. 

According to research, patients with OI have an increased risk of death compared with the general population not only due to respiratory failure but also due to gastrointestinal diseases and death following trauma. Furthermore, defects of collagen type 1 synthesis in OI patients increase the risk of cardiovascular diseases, including valvulopathies, atrial arrhythmias, and heart failure when compared with the general population [30]. 

In the presence of OI, normal skin structure and function might be disrupted. The skin changes associated with OI include thinness, translucency, easy bruisability, and impaired elasticity [31]. In this case report, epidermal necrosis of the hand might be associated with possible skin changes due to OI.

In our case, the features characteristic of severe OI were observed: multiple bone fractures and deformities, chronic respiratory failure complicated by periods of worsening due to infections, and development, motor, and growth delay. In the reviewed clinical cases, outcomes vary from severe disabling disease and death due to respiratory failure to disease with minimal and easily controlled symptoms. It shows that the severity of the disease depends on the type of OI.

The currently available treatment options are aimed at preventing new fractures, increasing bone mass, and increasing quality of life and function of the patient diagnosed with OI [28]. OI treatment options include a combination of non-surgical and surgical procedures. Non-surgical treatment examples include rehabilitation, bracing, splinting, and pharmacological management. Surgical interventions are recommended when needed. The most common medications used in OI treatment and new fracture prevention are bisphosphonates [32]. Bisphosphonates act by inhibiting osteoclast function. They also interact with osteoblasts and osteocytes. According to research, intravenous bisphosphonate treatment with pamidronate is associated with multiple benefits, including increased bone mass and mineral density and a decreased bone pain and fracture rate in patients diagnosed with severe OI [33]. Another medication used in patients with OI is denosumab [32]. Denosumab is a monoclonal antibody (IgG2) against receptor activator of nuclear factor kappa-B ligand (RANKL) that acts by inhibiting the formation, activation, and survival of osteoclasts without binding to the bone [34]. New treatment options are also being researched and are currently in the experimental stage. The future treatment of OI will focus on mesenchymal stem cell transplantation, genetic engineering, and the usage of molecular chaperones [32,35,36]. In our clinical case, the patient’s treatment is complex and non-surgical, including continuation of bisphosphonate therapy. The response to treatment is positive because no new fractures have been diagnosed for our patient (whose condition is severe but stable) since the start of bisphosphonate administration, whereas new fractures could lead to even more severe deformities and associated complications. In the reviewed cases, the treatment depends on the severity of the disease and ranges from conservative tactics to complex treatment, including treatment with bisphosphonates.

## 4. Conclusions

OI is a complex disease that, in severe cases, can be suspected before birth. OI cases require a multidisciplinary approach from the suspicion of the diagnosis due to possible diagnostic, treatment, and management challenges. In this clinical case, OI was suspected in the late second trimester during a detailed ultrasound examination and confirmed after birth. The correct suspicion of the diagnosis before birth led to successful delivery tactics and postpartum care. After birth, the newborn treatment was continued by a multidisciplinary team. Despite the severe form of the disease, it is currently managed. However, different types of OI lead to different severities for the disease and not all cases can be suspected before birth. In every case, if not suspected before birth, OI should be among the differential diagnoses when pathological bone fractures are observed immediately or soon after birth. 

## Figures and Tables

**Table 1:** Phenotypic features observed by the geneticist during newborn examination.

Body Part	Description
Whole body	Disproportionate
Skull	Uneven contour; mobile skull vault; fragmented skull bones; wide spaces between cranial sutures; wide anterior fontanel
Ears	Very low-set
Neck	Short
Intermammillary distance	Wide
Umbilicus	Low-set
Long bones	Very short humerus and femurs
Hands	Swollen
Feet	Clubfoot

**Table 2:** The main information from the case reports, including OI diagnosis before or soon after birth.

Year	Author	Familial Case	Ultrasound Findings	Phenotype Features after Birth	OI Type and Variant	Problems after Birth	Outcome
2022	Chang et al. [17]	No	US findings at 35 weeks of GA: Asymmetric IUGR, short and angulated long bones, breech position.	Dysmorphisms with brachycephaly; widely separated suture in the posterior aspect of the skull; triangular-shaped face; prominent forehead; blue and gray sclera; malar flattening; pointed chin; shortened and bowed extremities.	OI type III. Pathogenic variant in the *COL1A2* gene.	Respiratory distress; nutrition problems; prolonged apneic events associated with bradycardia and desaturations; pain.	Died from respiratory failure at day 30 of life.
2022	Luis et al. [18]	No	Not suspected before birth. Normal US findings.	Bilateral clavicle fractures with left brachial paresis. After one week: edema in the right hip joint; asymmetry in the folds; bilateral diaphyseal fracture of the femur.	OI type XV. Likely pathogenic variant c.324dup (p.(Gly109Argfs*46)) in probably homozygous state in the *WNT1* gene.	Bone fractures requiring hospitalization; pain.	–
2019	Canda et al. [19]	No	US findings at 17 weeks of GA: short, angulated, and bowed both femurs (< 5th percentile); shortened other limb bones.	–	OI type III. Pathogenic variant c.1588G>A (p.(Gly530Ser)) in the heterozygous state in the *COL1A1* gene.	–	Pregnancy termination at 23 weeks of gestation due to unfavorable prognosis.
2018	Bayram et al. [20]	No	Not suspected before birth. Normal US findings.	Not suspected after birth.	OI was confirmed with genetic testing. Detailed information about genetic testing and OI type was not provided.	Two weeks after birth: bilateral clavicle, bilateral femur, left humerus fractures.	No new fractures at the follow-up at 8 months old.
2021	Betoko et al. [21]	Case 1: No	Case 1: Not suspected before birth. Normal US findings.	Case 1: Not suspected after birth.	Case 1: OI type III was suspected according to clinical findings. Genetic testing not yet available in the country.	Case 1: At 26 days of age: reduced mobility; frontal bossing; moderate respiratory distress, bowed legs, shortened limbs. Multiple diaphyseal fractures of long bones; bones were curved and demineralized.	Case 1: At 9 months old: growth restriction; tiered vertebral collapse from T12 to L3 with no spinal deformities. Treatment with bisphosphonates prescribed.
2021	Betoko et al. [21]	Case 2: No	Case 2: US findings at the third pregnancy trimester: bowing and shortening of both femurs.	Case 2: Deformed limbs.	Case 2: OI type II or III was suspected according to antenatal and clinical findings. Genetic testing not yet available in the country.	Case 2: Neonatal sepsis at the age of 2 days. At 37 days of age: reduced mobility; frontal bossing; blue sclerae; bowed legs; shortened limbs. Multiple diaphyseal fractures of the long bones; fracture of the sternum; bones are curved.	Case 2: At the age of 3 months: pain improved; bone callus formed. Oral vitamin D supplements prescribed.
2020	Wu et al. [22]	No	Not suspected before birth. Normal US findings.	Not suspected after birth.	OI type V. Pathogenic variant c.-9C>A in heterozygous state in the *IFITM5* gene.	2 days after birth: pain and irritability. A transverse right mid-clavicular fracture was observed in a chest X-ray.	No new fractures and healed clavicle fracture at the age of 1 month old.
2018	Celin et al. [23]	No	US findings at 30 weeks of GA: severe polyhydramnios; delayed skull ossification; shortening and bowing of the long bones.	Blue sclera; hypoplastic thorax; bowing of the limbs; multiple fractures that occurred in utero (fractures of ribs, clavicles, humerus, upper limbs, and multiple compression fractures of spine).	OI type V. Pathogenic variant c.119C>T (p.(Ser40Leu)) in the heterozygous state in the *IFITM5* gene.	Multiple bone fractures; transient tachypnea of the newborn; pain; nutritional problems.	At the age of two: development and motor delay; blue sclera; dentinogenesis imperfecta; short stature; bowed and short limbs; deformed thorax; relative macrocephaly with wide anterior fontanel. At age of 5: scoliosis, worsening of thoracic and skull deformations.
2019	Alhousseini et al. [24]	No	US findings at 21 weeks of GA: shortened and bowed femurs; both humerus with old fractures and callus; both tibia and fibula bowed.	Neonatal X-ray revealed: new and old healed fractures of extremities including the femur bones and humerus. Undermineralized extremities.	OI type II. Pathogenic variant c.1840G>C (p.(Gly614Arg)) in heterozygous state in the *COL1A1* gene.	Respiratory distress requiring oxygen therapy; pain.	At the age of 22 months: mild bilateral conductive hearing loss; no new fractures or seizures since birth; dentinogenesis imperfecta; short and bowed limbs; able to sit on her own; not able to walk.

Abbreviations: US—ultrasound; GA—gestational age; IUGR—intrauterine growth restriction.

## Data Availability

Data sharing is not applicable to this article.
